# Environmental Controls Over Actinobacteria Communities in Ecological Sensitive Yanshan Mountains Zone

**DOI:** 10.3389/fmicb.2016.00343

**Published:** 2016-03-22

**Authors:** Hui Tang, Xunxun Shi, Xiaofei Wang, Huanhuan Hao, Xiu-Min Zhang, Li-Ping Zhang

**Affiliations:** ^1^College of Life Sciences, Hebei UniversityBaoding, China; ^2^The Key Lab of Microbial Diversity Research and Application of Hebei ProvinceBaoding, China; ^3^Key Laboratory of Medicinal Chemistry and Molecular Diagnosis, Ministry of Education, Hebei UniversityBaoding, China

**Keywords:** ecological sensitive zone, a Yanshan mountains, Actinobacteria, phylogenetic diversity, 16S rRNA Actinobacterial clone library

## Abstract

The Yanshan Mountains are one of the oldest mountain ranges in the world. They are located in an ecologically sensitive zone in northern China near the Hu Huanyong Line. In this metagenomic study, we investigated the diversity of Actinobacteria in soils at 10 sites (YS1–YS10) on the Yanshan Mountains. First, we assessed the effect of different soil prtreatment on Actinobacteria recovery. With the soil pretreatment method: air drying of the soil sample, followed by exposure to 120°C for 1 h, we observed the higher Actinobacteria diversity in a relatively small number of clone libraries. No significant differences were observed in the Actinobacterial diversity of soils from sites YS2, YS3, YS4, YS6, YS8, YS9, or YS10 (*P* > 0.1). However, there were differences (*P* < 0.05) from the YS7 site and other sites, especially in response to environmental change. And we observed highly significant differences (*P* < 0.001) in Actinobacterial diversity of the soil from YS7 and that from YS4 and YS8 sites. The climatic characteristics of mean active accumulated temperature, annual mean precipitation, and annual mean temperature, and biogeochemical data of total phosphorus contributed to the diversity of Actinobacterial communities in soils at YS1, YS3, YS4, and YS5 sites. Compared to the climatic factors, the biogeochemical factors mostly contributed in shaping the Actinobacterial community. This work provides evidence that the diversity of Actinobacterial communities in soils from the Yashan Mountains show regional biogeographic patterns and that community membership change along the north-south distribution of the Hu Huanyong Line.

## Introduction

Microbial communities can diverge rapidly, and result in distinct biogeographic patterns (Green et al., 2008). However, based on different evolution, biogeographic patterns are posited to consist of dramatic range expansion as a result of effect at the genotype level (Ramette and Tiedje, 2007). For microbial biogeography, the traditional view has been that “Everything is everywhere, but the environment selects” (Baas, 1934). There has been a debate over whether variation in microbial communities through space results from environmental, or from geographic barriers and other human activities that contribute to community structure (Eisenlord et al., 2012). If not all microbes are evenly dispersed over time, this would suggest that forces structuring the microbial communities are more complex than only adaptive evolution via natural selection (Bissett et al., 2010; Kuang et al., 2015; Yang et al., 2015). We addressed this issue by examining the community structure of a deeply diverse and divergent phylum, the Actinobacteria. Actinobacteria are important organisms that mediate plant litter decay and the subsequent formation of soil organic matter in terrestrial ecosystems. This phylum is phylogenetically divergent and the closest prokaryotic relative is yet to be identified (Ventura et al., 2007; Mendes et al., 2015). Actinobacteria express a variety of morphologies and life-history traits that could be advantageous for dispersal, including sporulation. Here, we evaluated by metagenomic technology whether environmental disturbance of an ecologically sensitive zone is associated with a highly structured community of soil Actinobacteria in the Yanshan Mountains of northern China. Microorganisms are the most diverse and abundant group of organisms on Earth; however, in soil microbial communities, work to understand this diversity has been primarily directed toward general rather than group-specific diversity. Actinobacteria, ubiquitously found in terrestrial (Han et al., 2015), freshwater (Mullowney et al., 2015), and marine (Sun et al., 2015) ecosystems, are the dominant soil bacterial phylum and they are believed to play multiple roles in the environment (Barka et al., 2016).

The construction of metagenomic libraries and other DNA-based metagenomic projects are initiated by the isolation of high-quality DNA that is suitable for cloning and that covers the microbial diversity present in the original sample. Since Pace et al. (1985) first proposed the direct cloning of environmental DNA, soil DNA extraction techniques, including both direct and indirect methods (Robe et al., 2003; Delmont et al., 2010), have been developed. These efforts have led to the development of various homemade DNA extraction protocols, as well as commercial kits, which have been used in more than 1000 studies reported yearly. Therefore, high quality DNA has been isolated from a variety of environments. In addition, cultivation-independent methodologies, particularly sequence analyses of cloned 16S ribosomal RNA genes (16S rDNA) are powerful tools to investigate microbial diversity. Most approaches target the 16S rRNA gene for PCR amplification and subsequent Sanger sequencing of the clone libraries (Sogin et al., 2006), ribosomal sequence tags (SARST; Poitelon et al., 2009), denaturing gradient gel electrophoresis (DGGE; Yim et al., 2015), terminal restriction fragment length polymorphism (T-RFLP; Lazzaro et al., 2015), Pyrosequencing (Schäfer et al., 2010), or 454 Life Sciences and Illumina analyses (Vasileiadis et al., 2012; Logares et al., 2014). However, there is no specific primers for Actinobacteria to construct a full or near full-length 16S rDNA clone libraries. And the Actinobacterial-specific primers used for high-throughput technique can obtain some information of Actinobateria, but sometimes the recovered sequence is too small to gain complete genetic information and detailed phylogenetic characterization of Actinobacteria, especially for a greater number of unclassified Actinobacteria.

Therefore, in this study, it was purpose to obtain a full or near full-length 16S rDNA sequence of Actinobacteria. To increase the proportion of Actinobacteria in the 16S rDNA library, we developed a method of soil pretreatment to concentrate the Actinobacterial community, and used a PCR primer system to capture Actinobacteria from prokaryotes in the 16Sr DNA full-length clone library. The purpose of the present study was to compare the community structure and phylogenetic diversity of Actinobacteria among various sites in the Yanshan Mountains.

## Methods

### Sample collection

**Figure 1:**
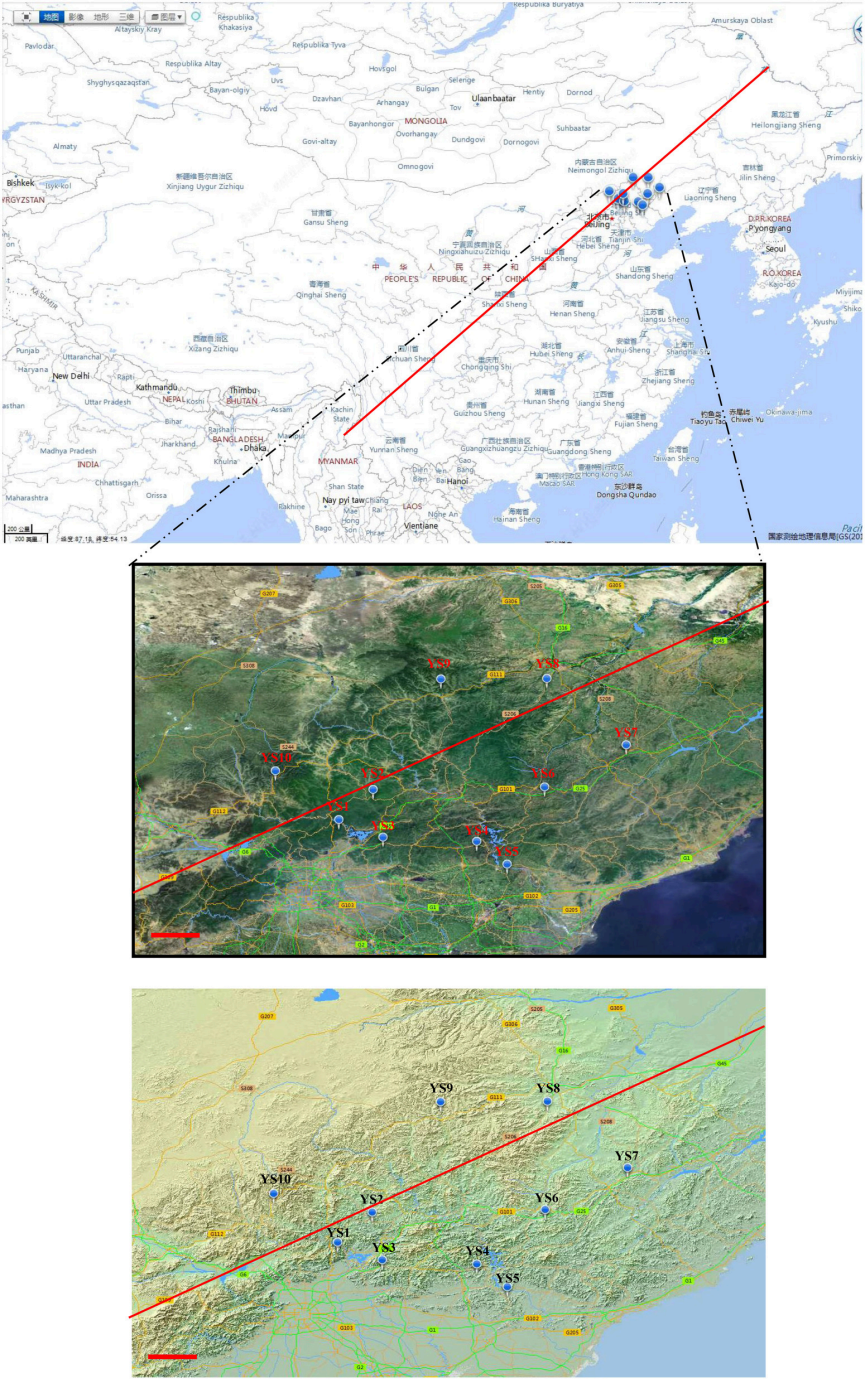
**Various sampling points along ecological sensitive Yanshan mountains zone**. The circular mark denotes the stations. The photograph and topographic map were provided by the Mapword (http://map.tianditu.com/map/index.html. Red line is Hu Huanyong Line).

Soil samples were collected from various locations in the Yanshan Mountains (Figure 1) on October 2–10, 2011. Descriptions of soil collection sites are presented in Table 1. In each of the 10 sites, there were 3 randomly selected 30 m × 30 m replicate plots 100–150 m apart. In each plot, we collected 10 soil samples using a 2.5 cm diameter soil core, which extended to a depth of 10 cm. The 10 soil samples in each plot were composited and passed through a 2-mm sieve in the field. By pooling the 10 soil cores, we aggregated spatial heterogeneity at the scale of individual plots. The 3 soil plot samples were combined into a representative sample for each site. From the sieved composite sample, a 5.0-g sample was removed for DNA extraction. This was done to allow a characterization of the Actinobacteria community at the scale of the entire Yanshan Mountains, and to explore regional trends in community similarity that may have been structured by environmental factors.

**Table 1:** **Actinobacteria sequencing statistics and α diversity measures of different pretreatment of soil samples**.

**Source^a^**	**Total no. of clones**	**Detection rate (%)**	**OTU^b^**	**Shannon diversity index (H^∕^ (loge))**	**Equitability_J**	**Buzas and Gibson's evenness (E) index**
A1	102	20.4	74	4.161	0.9668	0.866
A2	30	6.0	19	2.731	0.9674	0.807
A3	73	14.6	64	4.101	0.9762	0.943
B1	44	8.8	38	3.595	0.9783	0.958
B2	28	5.6	26	3.233	0.9724	0.975
B3	84	15.8	61	4.020	0.9779	0.913
C1	55	11.0	37	3.464	0.9693	0.863
C2	29	5.8	23	3.062	0.9767	0.929
C3	24	4.8	20	2.925	0.9765	0.931
CK	18	3.6	15	2.659	0.9720	0.952

a *Sources of data are from the following libraries: uncultured Actinobacteria are from treated samples A1, A2, A3, B1, B2, B3, C1, C2, C3, and CK*.

b *OUT were defined as clone sequences with < 97% 16S rRNA gene sequence similarity to other clones*.

### DNA extraction methods

We design three kinds of soil pretreatment method to improve the proportion of Actinobacteria DNA. (i) For protocol A, air dried soil sample were treated by 120°C 1 h (A1), 2 h (A2), 3 h (A3) respectively; (ii) For protocol B, soil sample were treated by air drying processing 15 days (B1), 30 days (B2), 45 days (B3) respectively; (iii) For protocol C, soil sample were treated by 0.1% Polymyxin B Sulfate immersion 1 h (C1), 2 h (C2), 3 h (C3) respectively; After pretreatment of soil samples, and centrifugal washing three times with sterile water for removing DNA of release, DNA extraction from 1.0 g soil samples was carried out using the PowerSoil™ DNA Isolation Kit (Mo Bio Laboratories), according to the manufacturer's instructions. The yield and integrity of the environmental DNA obtained were confirmed through electrophoresis in 1% agarose gel.

### Construction of 16S rRNA gene libraries

The purified DNA was used as a template to specifically amplify 16S rRNA gene fragments, a ~1500 bp region using the bacteria-specific primers (Lane, 1991): 27F (5-AGAGTTTGATCC/ATGGCTCAG-3) and 1525R (5-AAGGAGGTGA/TTCCAA/GCC-3). To recondition the PCR product for elimination of heteroduplexes in mixed-template PCR (Janelle et al., 2002), the amplified reaction was diluted 10-fold into a fresh reaction mixture of the same composition and cycled three times. The size and quality of the resulting PCR products was confirmed by agarose gel electrophoresis (1.4% agarose). They were then cloned into the pUCm-T linear plasmid vector (Takara Bio Group, Code D101A) and then into *E. coli* DH5a competent cells (Takara Bio Group). After the transformants were grown overnight, single-clone colonies were picked up with sterile toothpicks and transferred into 1.5 mL microcentrifuge tubes containing 50 mL of TE buffer. The tubes were heated for 15 min at 95 C to lyse the cells, and then chilled on ice. Insert 16S rDNA sequences were identified by M13/pUC sequencing primer and M13/pUC reverse primer (approximately 1.5 kb).

### Amplification and sequencing of actinobacteria *16S rRNA* genes

Two different Actinobacteria-specific primer sets specifically targeting 16S rRNA gene were used to confirm the presence of selected Actinobacteria genotypes in soil DNA. The first primer set, Com2xf /Ac1186 (Schäfer et al., 2010), was used to detect most Actinobacteria species. The 25-μL PCR reaction mixture contained 2.5 μL PCR buffer, 2 μL MgCl_2_ (25 mM), 2 μL dNTPs (2.5 mM), 0.5 μL each primer (10 μM, Shenggong Biotech, Shanghai, China), 17.7 μL H_2_O, 0.2 μL BSA (20 mg/mL^−1^), and 0.1 μL Taq polymerase (5 U/μL^−1^) (Takara, Japan). This mixture was added directly to cloned cells. PCR was carried out in a thermocycler (Bio-Rad, München, Germany) with an initial denaturation step at 95°C for 10 min, followed by 25 cycles of 30 s at 94°C, 30 s at 60°C, and 30 s at 72°C, followed by a final extension at 72°C for 5 min. A second PCR using the primer set SC-Act235-aS-20/SC-Act878-aA-19 (Stach et al., 2003) was carried out to increase the amount of detectable Actinobacteria DNA. The 25-μL reaction mixture contained 2.5 μL PCR buffer, 2.5 μL MgCl_2_ (25 mM), 2 μL dNTPs (2.5 mM), 0.5 μL each primer (10 μM, Shenggong Biotech, Shanghai, China), 17.7 μL H_2_O, 0.2 μL BSA (20 mg/mL^−1^), and 0.1 μL Taq polymerase (5 U/μL^−1^) (Takara, Japan). The reaction mixture was also added directly to cloned cells. PCR was performed with an initial denaturation step at 95°C for 10 min, followed by 25 cycles of 30 s at 94°C, 30 s at 60°C, and 1 min at 72°C, followed by a final extension at 72°C for 5 min. The success of PCR reactions were determined by subjecting the amplified products to 1% agarose gel electrophoresis and ethidium bromide staining. All positive clones and the A3 clone library were recultured in LB broth, and sequenced using Shenggong Biotech, Shanghai, China.

### Phylogenetic analyses

The 16S rRNA gene sequences were taxonomically assigned using the Naïve Bayesian rRNA classifier of the Ribosomal Database Project II (RDP; Wang et al., 2007). Sequences from this study were subsequently aligned using the ClustalW multiple alignment tool from BioEdit v7.0.5.3. The program DNADIST v3.5c in BioEdit was used to compute a distance matrix from the aligned nucleotide sequences. The distance matrix was input into the DOTUR program (v1.53) to assign the sequences to operational taxonomic units (OTUs) using the furthest-neighbor clustering algorithm (Schloss and Handelsman, 2005) at 97, 95, and 90% identities. Sequences from each clone library were aligned separately, and OTUs were identified at 97% identity. One representative sequence was selected for each OTU. Representative sequences from each OTU (97%) in 10 libraries determined in this study were deposited in the NCBI database under accessions no. KC554071–KC554721. Coverage (C) was used as a measure of captured diversity, where C is expressed by 1_n1/N, in which n1/N is the ratio of the number clones that appeared only once (n1) to the total number of clones (N). Rarefaction curves were produced by standard calculations by comparing the total number of clones obtained to the number of clones representing unique OTUs. Sampling sufficiency of each library was determined as described by Kemp and Kemp and Aller (2004) using the “Large Enough” estimator available online at http://www.aslo.org/lomethods/free/2004/0114a.html. The Shannon index, Simpson's diversity index, and nonparametric richness estimators ACE and Chao1 were calculated using the DOTUR program (Schloss and Handelsman, 2005). A neighbor-joining tree was created using MEGA version 4 software. The bootstrap values represent 1000 samplings. Multiple environments were simultaneously analyzed using phylogenetically comparing the microbial communities using weighted and unweighted UniFrac to conduct a principal coordinates analysis (Lozupone et al., 2006). The neighbor-joining tree generated for input to UniFrac was limited to 999 sequences. The environmental input file for UniFrac contained a count of how many times the selected sequence appeared in the clone library. The UniFrac significance test with abundance weights was used to determine significant differences in the Actinobacteria community structure. *P*−values were corrected for multiple comparisons by multiplying the calculated *P*−value with the number of comparisons made (Bonferroni correction; Lozupone et al., 2006).

### Environmental variables and multivariate statistical analysis

Environmental characteristics were assembled into two data sets: (1) a biogeochemical data set composed of factors, and (2) climatic characteristics. The biogeochemical data matrix included soil pH and total nitrogen (TN); total phosphorus (TP); available phosphorus (AP); available potassium (AK); organic matters (OM) (Supplementary Table 1). The second matrix characterized climatic variation by including annual mean temperature (MT); annual mean precipitation (MP); mean sea level elevation (ME); annual mean sunshine duration (SD); mean active accumulated temperature (>10°C) (AAC) (Supplementary Table 2). The climatic data used in this study were averages from the years 1981 to 2012. Environmental vectors, of biogeochemical and climatic data sets, were fit to nMDS ordinations of biological data, which identified the individual variables correlated with community patterns. Redundancy analysis (RDA) was used to examine the correlations between species patterns and environmental variables to evaluate which variables explained significant proportions of variation in Actinobacteria community composition. Additional statistics were conducted in the R package vegan (Oksanen et al., 2011).

## Result

### Testing of an actinobacteria primer system

The Actinobacteria specific primer systems detected 75 positive clones from the 16S rDNA clone library of the A3 sample. To determine the validity and specificity of the primer system, all clones in the library were sequenced and classified. Two out of 75 positive clones and another 425 clones belonged to the Acidobacteria, Proteobacteria and Firmicutes, and 73 positive clones were Actinobacteria belonging to 20 known and 34 unknown genera.

### Effect of different soil pretreatments on actinobacteria recovery

**Figure 2:**
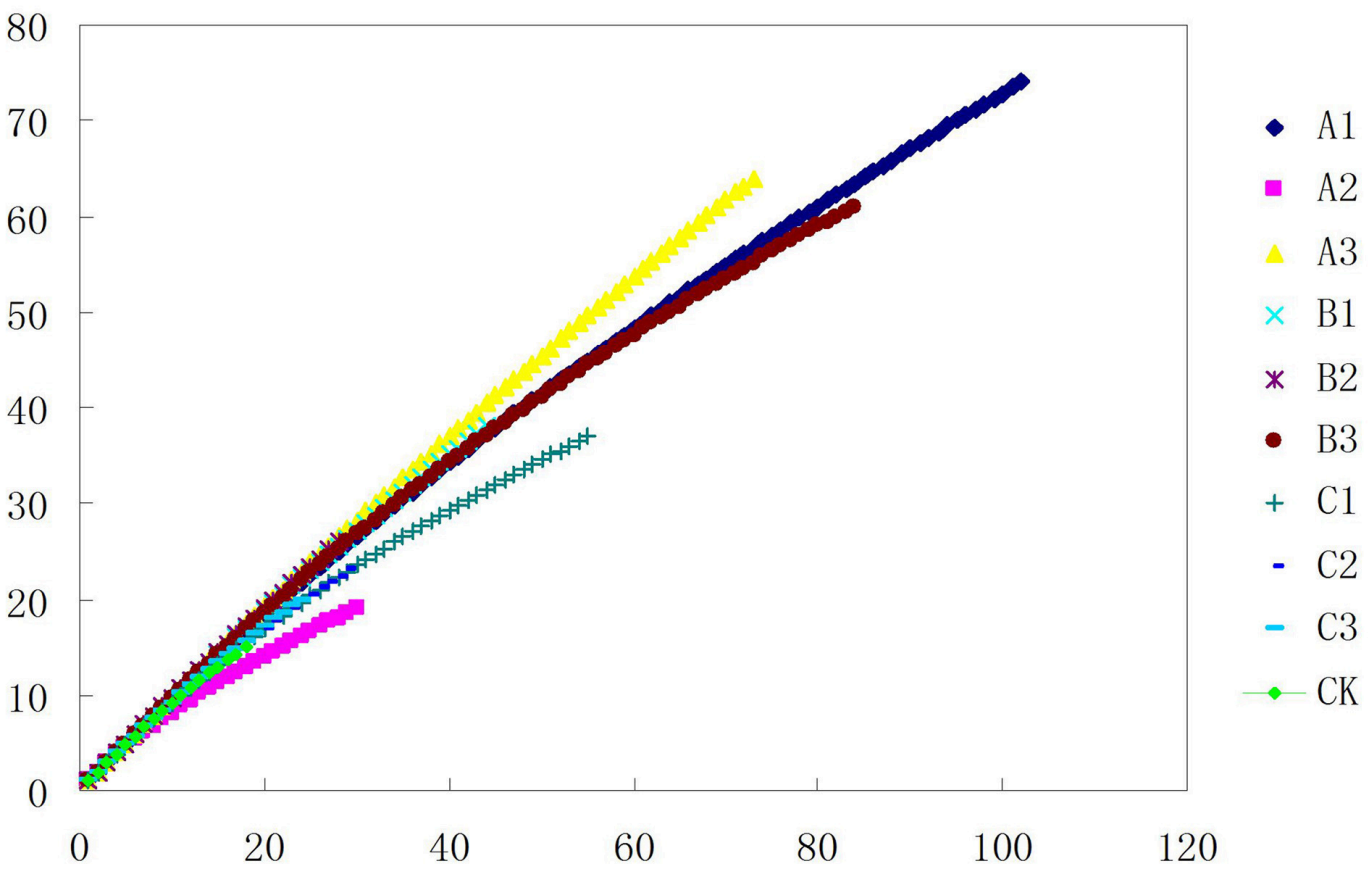
**Phylotype richness curves for clone and culture libraries**. Sampling curves were calculated by rarefaction^56,57^.

**Figure 3:**
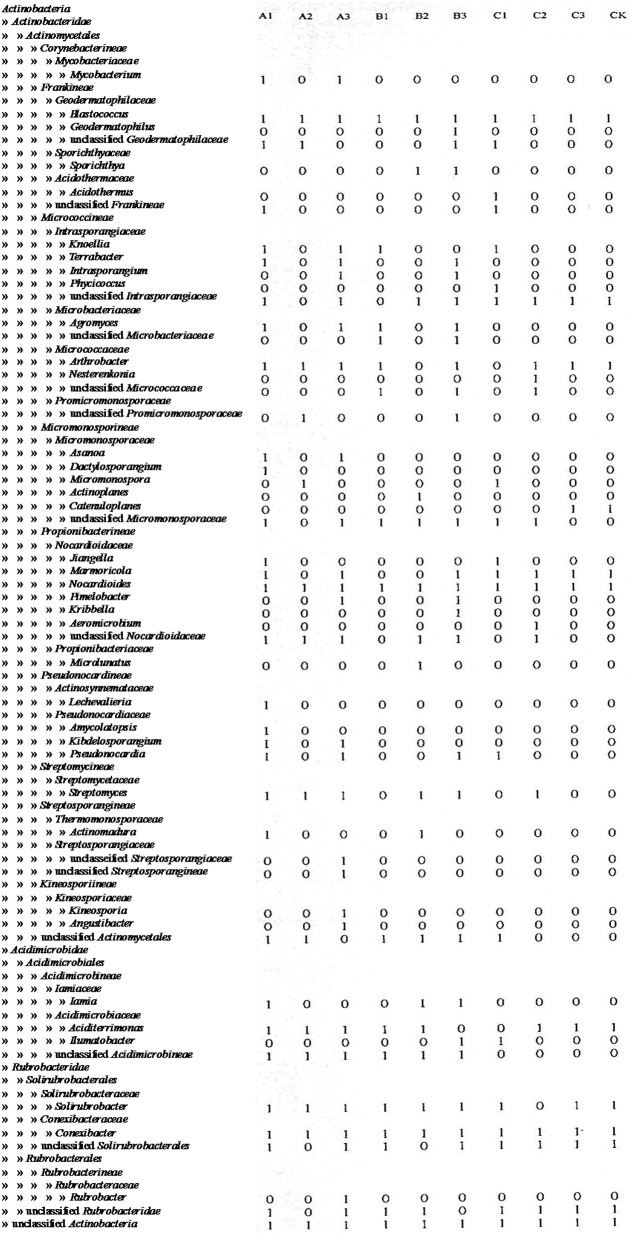
**Distribution of Actinobacteria clones from different pretretment in taxonomy**.

**Figure 4:**
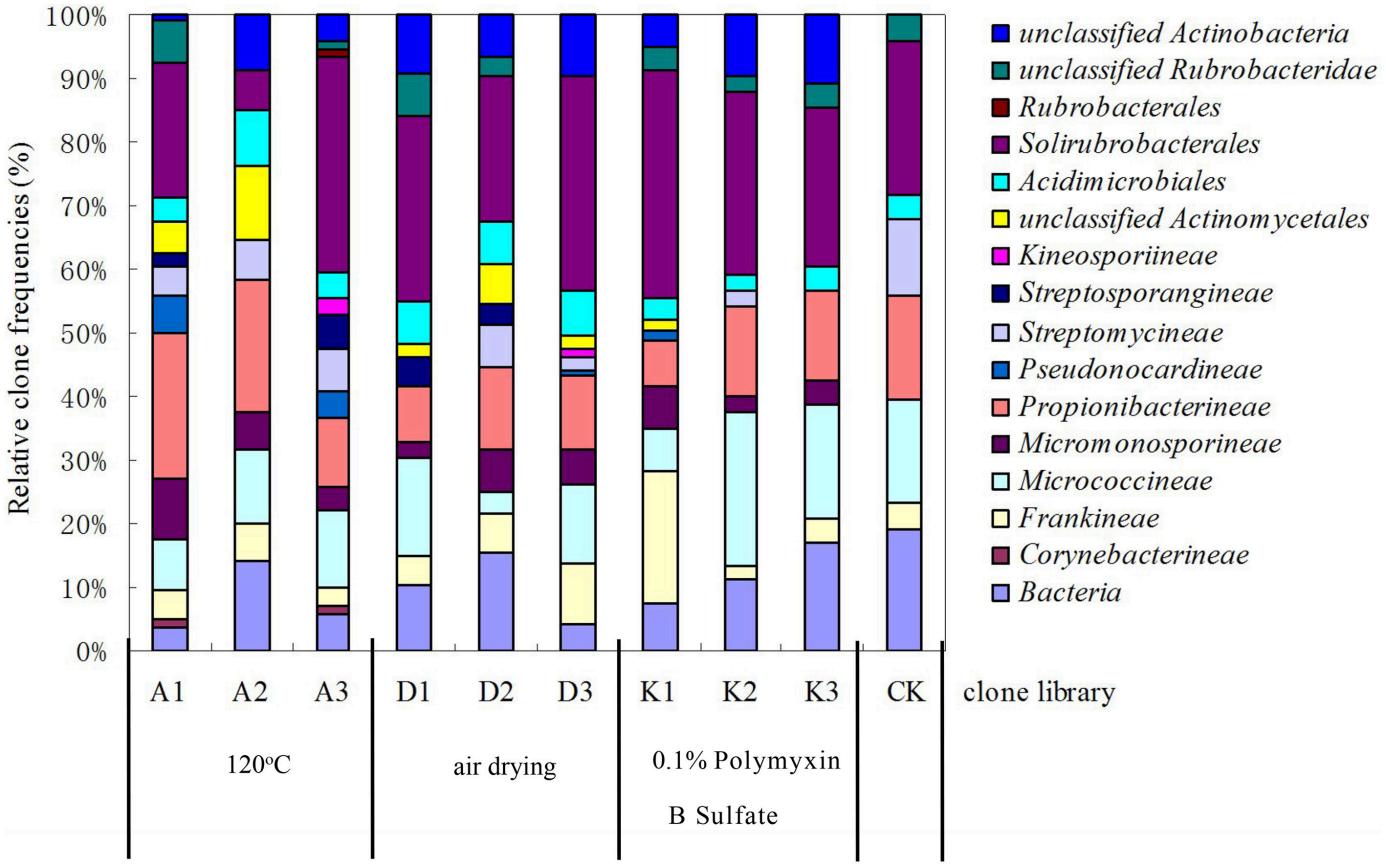
**Relative clone frequencies in major phylogenetic groups of the clone libraries from different pretreatment of soil sample**.

After soils were pretreated, we detected a larger number and phylotype s of Actinobacteria in the same numbers of prokaryotic microorganisms in the 16S rDNA cloned library (Table 1). It were corroborated by the diversity indices, which were significantly higher than direct extraction of Kit (CK). In addition, the number Actinobacteria clones detected was significantly different among samples treated by the 3 pretreatment methods. The total detection rate using each of these methods were: A (13.7%) > B (10.4%) > C (7.2%). Protocol A1 yielded 102 clones with a 20.4% detection rate (from 500 clones); protocol B3 yielded 84 with 15.8% detection; protocol A3 yielded 73 with 14.6% detection; CK yield 18 with 3.6% detection. Sequences with 97% similarity in the 16S rRNA gene used for phylogenetic analyses were combined into OTUs. A total of 252 OTUs were present in the 10 clone libraries. Most of them were A1 (74 out of 102 clones), next were A3 (64 out of 73 clones), third were B3 (62 out of 84 clones), while CK had only 15 (out of 18 clones). In addition, only A1 contained all OTUs recovered by CK. Even if the rarefaction curves did not approach an asymptote (Figure 2), meaning that we did not capture the full diversity of the Actinobacterial community, 10 clones representing 37 known genera out of a total of 186 genera were detected, with A1 yielding 21 (out of 56) known genera; A3 yielding 20 (out of 54) genera; B3 yielding 17 (out of 54) genera. They were all far more than CK, 8 out of 12 genus. *Nocardioids* and *Conexibacter* and some unclassified groups were detected in the 10-clone library. Unique known genera detected using the A1 method were *Dactylosporangium, Lechevalieria*, and *Amycolatopsis*; A3 resulted in the detection of *Kineosporia* and *Angustibacter*; B2, *Microlunatus* and *Actinoplanes*; B3, *Geodermatophilus* and *Kribbella*; C1, *Acidothermus* and *Phycicoccus*; and C2, *Nesterenkonia* and *Aeromicrobium*. The A2, B1, and C3 had not unique known genus but unclassified group (Figure 3). The Actinobacterial compositions at the order/suborder levels were significantly different between the pretreated or untreated soil samples (Figure 4). The pretreated soil allowed increased detection of specific orders/suborders, including *Solirubrobacterales, Propionibacterineae, Frankineae, Acidimicrobiales*, and *Micrococcineae*. However, *Corynebacterineae, Kineosporiineae*, and *Rubrobacterales* were only detected in the A3 library, which clearly indicated that pretreatment of soil could lead to an underestimation of some Actinobacteria groups. Furthermore, the Actinobacterial library was dominated by the *Solibubrobacterales* (A2, 6.8%; C1, 38.2%) of the Actinobacteria clones and A2, 0.4%; B3,−6.4% of all 16S rDNA clones, whereas *Propionibacterineae* dominated the A1 and A2 libraries (24.0% of the Actinobacterial clones and 24.1% of all 16S rDNA clones; Figure 4). The B3 library allowed the detection of a greater number of unclassified Actinobacteria, unclassified *Rubrobacteridae*, and unclassified *Actinomycetales* than the A1 library.

### Actinobacteria community composition at stations in the Yanshan Mountains

Soil samples from 10 stations were treated at 120°C for 1 h, then the bacterial 16S rDNA clone library was constructed. We randomly selected 1000 clones (for sufficient Actinobacterial coverage) from each station to detect Actinobacteria using 2 Actinobacteria-specific primer sets. From the 10,000 clones generated, approximately 13% (*n* = 1327) resulted in PCR products from the Actinobacteria-specific primers.

**Figure 5:**
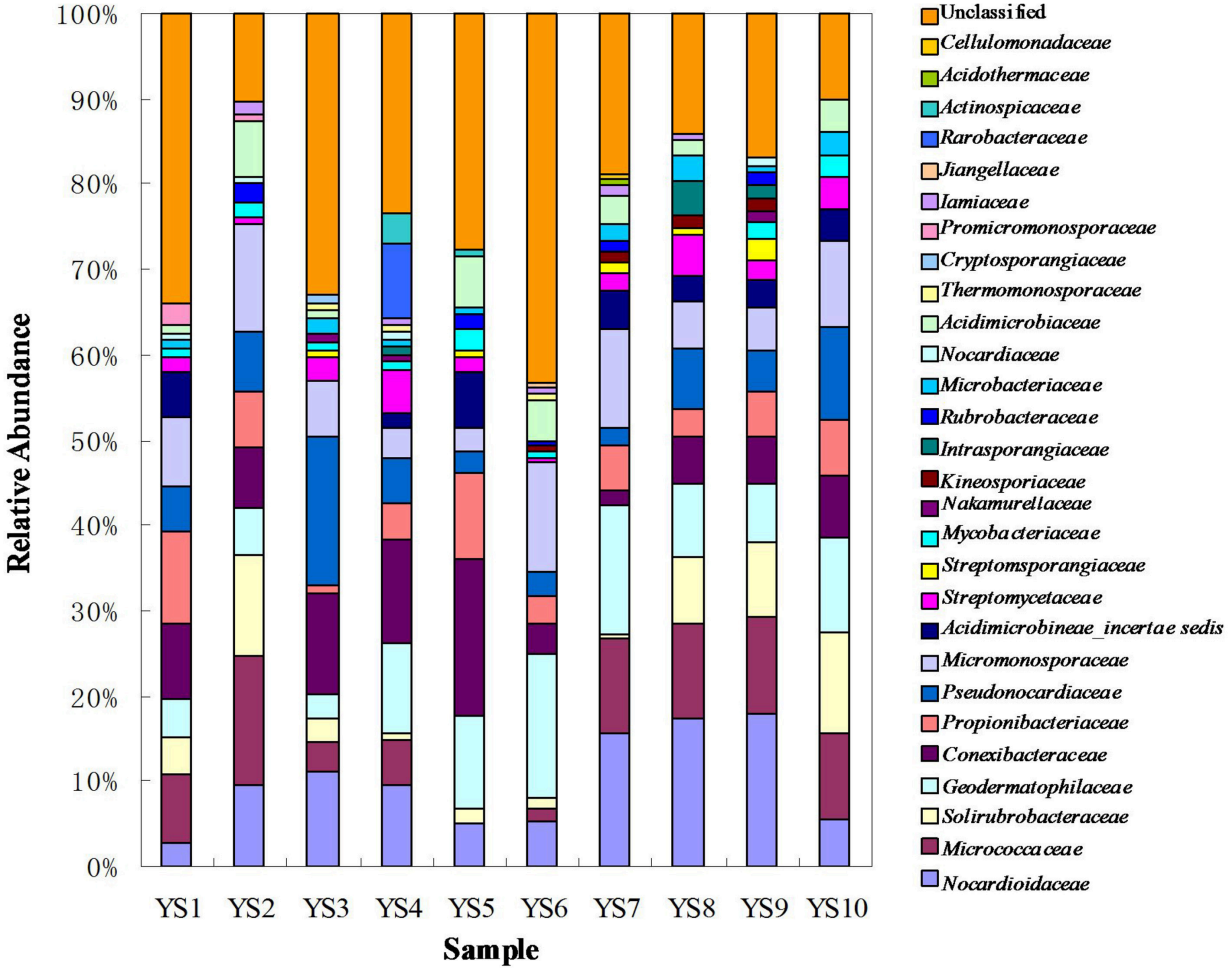
**Composition of different family based on classification of 16S rRNA sequences of Actinobacteria from soil of ten sites**.

Depending on the station surveyed, the proportion of Actinobacteria among total clones varied between 10.8 and 20.4% (Table 2), and resulted in 575 OTUs grouped at the 97% similarity level. The “Large Enough” calculator was used to determine whether individual clone libraries were sampled sufficiently. If the estimated phylotype richness reached an asymptote, we inferred that the library was large enough to yield a stable estimate of phylotype richness. According to the figure, all sites appeared to have been sufficiently sampled (Supplementary Figure 1). We identified OTUs in 28 of 39 Actinobacterial families, classified by the RDP (Figure 5). For the most abundant OTUs, the closest similarity to known organisms was 100% to members of the *Blastococcus* genus, *Frankineae* family. UniFrac metrics were used to assess community similarity between 2 or more samples according to their structure (weighted/quantitative) and membership (unweighted/quantitative). In the 2-dimensional plot visualized by the UniFrac weighted distance matrix principle coordinates analysis (3% dissimilarity), the samples of each system distinctively responded to the majority of the variation detected in the samples across 2 axes (Figure 7A). Axis 1 accounted for 21.82% of the variation, and Axis 2 accounted for 19.29% of the variation. In Figure 7B, the same 2-dimensional plot was shown for the unweighted method, which showed that samples from the same type, were in consideration of community membership, although less distinctive (Axis 1 = 16.72%, Axis 2 = 13.14%). The results from the UniFrac weighted and unweighted PCA plots demonstrate distinctions in structure and composition of the Actinobacterial communities from different stations. Furthermore, the UniFrac significance test results revealed significant differences in community membership between sites YS1 and YS6 (*P* < 0.001), sites YS4 and YS7 (*P* < 0.001), and sites YS7 and YS8 (*P* < 0.001) (Supplementary Figure 2). Diversity estimates, Ace and Chao1, indicated that YS6, YS7, and YS9 were more diverse than the other sites.

**Table 2:** **Actinobacteria sequencing statistics and α diversity measures of soil samples of Yanshan mountains zone**.

**Source^a^**	**Total no. of clones**	**OTU^b^**	**Shannon diversity index (H^∕^)**	**Pielou's evenness (J') index**	**Chao 1**	**ACE**
YS1	114	76	4.127	0.9529	198.04	255.21
YS2	125	54	3.563	0.8932	161.51	154.02
YS3	108	77	4.151	0.9557	171.64	321.31
YS4	115	65	3.869	0.9269	159.91	209.37
YS5	121	59	3.741	0.9275	106.86	142.94
YS6	149	66	3.771	0.9001	466.81	200.04
YS7	149	107	4.459	0.9543	373.64	579.72
YS8	129	62	3.834	0.9290	126.77	131.93
YS9	204	138	4.713	0.9565	498.87	548.14
YS10	113	51	3.495	0.8889	80.75	126.56

*Conexibacteraceae, Geodermatophilaceae, Micrococcaceae, Micromonosporaceae, Nocardioidaceae, Propionibacteriaceae, Pseudonocardiaceae*, and *Solirubrobacteraceae* represented 46.4–66.9% of the bacterial community in each station. These taxa together accounted for an average of 55% of the Actinobacterial clones obtained from soil of the 10 stations in the Yanshan Mountains. *Geodermatophilaceae, Micromonosporaceae, Nocardioidaceae, Propionibacteriaceae, Pseudonocardiaceae, Streptomycetaceae*, and *Solirubrobacteracea*e were common to the 10 libraries, and they were identified as contributing substantially to the relative abundance of Actinobacteria (Figure 5). To demonstrate the differences in Actinobacterial community composition, relative abundances of Actinobacteria were also assessed. Table 3 displays the relative abundances and Shannon diversity indices of the salient families of Actinobacteria identified in the soils from the 10 stations. Groups of family, YS10 were fewest, YS4 were most. The UniFrac metric identified the unique phylogenetic branch belonging to Actinobacterial communities within each site compared to the entire community (*P* = 0.001). The unique family of site YS3 was *Cryptosporangiaceae*, YS4 was *Rarobacteraceae*, and YS6 was *Jiangellaceae*, and the unique families of YS7 were *Acidothermaceae* and *Cellulomonadaceae*.

**Table 3:** **Abundance and diversity of main family from Actinobacteria**.

	**YS1**		**YS2**		**YS3**		**YS4**		**YS5**		**YS6**		**YS7**		**YS8**		**YS9**		**YS10**	
	**RA^a^**	**SI^b^**	**RA**	**SI**	**RA**	**SI**	**RA**	**SI**	**RA**	**SI**	**RA**	**SI**	**RA**	**SI**	**RA**	**SI**	**RA**	**SI**	**RA**	**SI**
*Nocardioidaceae*	2.1	1.39	8.5	1.77	8.5	1.91	7.8	1.91	4.2	1.33	5.7	1.56	4.3	2.20	17.0	2.82	15.6	1.91	26.2	3.02
*Micromonosporaceae*	8.7	1.58	15.4	1.06	6.7	1.95	3.8	1.39	2.9	1.10	18.3	1.77	10.6	1.70	17.3	2.51	6.7	1.55	9.6	2.39
*Conexibacteraceae*	9.7	1.75	8.7	1.87	12.6	2.49	13.6	1.58	21.4	1.64	4.9	1.73	7.8	1.61	2.9	0.69	6.8	0.00	11.7	2.06
*Pseudonocardiaceae*	7.4	1.79	11.1	0.00	23.5	1.32	7.4	1.56	3.7	1.10	4.9	1.10	14.8	0.50	3.7	1.10	11.1	1.58	12.4	2.04
*Propionibacteriaceae*	16.4	1.10	11.0	0.74	1.4	0.00	6.8	1.33	16.4	0.72	6.8	0.60	9.6	0.50	11.0	1.39	5.5	0.00	15.1	2.03
*Acidimicrobineae_incertae_sedis*	15.8	1.79	0.0	0.00	0.0	0.00	5.3	0.69	21.1	0.41	0.0	0.69	10.5	0.00	18.4	1.33	10.5	0.00	18.4	1.75
*Micrococcaceae*	8.5	1.00	17.9	0.69	3.8	1.39	5.7	0.87	0.0	0.00	1.9	0.38	10.4	0.64	16.0	1.66	13.2	0.69	22.6	1.71
*Geodermatophilaceae*	4.0	0.50	5.6	0.41	2.4	1.10	9.6	0.84	10.4	0.91	20.0	0.43	9.6	1.44	18.4	1.62	8.8	1.30	11.2	1.43
*Streptomycetaceae*	6.1	0.69	3.0	0.00	9.1	0.64	18.2	0.87	6.1	0.69	3.0	0.64	12.1	0.69	9.1	0.00	18.2	0.69	15.2	1.39
*Solirubrobacteraceae*	7.1	1.61	21.4	0.41	4.3	0.00	1.4	0.00	2.9	0.69	2.9	0.69	18.6	0.69	1.4	0.00	14.3	1.23	25.7	1.21
*Streptosporangiaceae*	0.0	0.00	0.0	0.00	10.0	0.00	0.0	0.00	10.0	0.00	0.0	0.00	0.0	0.00	20.0	0.69	10.0	0.00	50.0	0.95
*Mycobacteriaceae*	6.2	0.00	12.5	0.64	6.3	0.00	6.3	0.00	18.7	0.64	6.2	0.64	18.8	0.69	0.0	0.64	0.0	0.00	25.0	0.56
*Acidimicrobiaceae*	2.9	0.00	22.8	0.74	2.9	0.00	0.0	0.00	20.0	0.41	20.0	1.04	11.4	0.41	14.3	1.61	5.7	0.69	0.0	0.00
*unclassdified*	12.5	3.05	4.3	2.51	11.8	3.14	8.9	2.65	10.9	2.91	21.0	1.85	3.6	2.54	9.6	3.15	5.9	2.29	11.5	3.26

a *Relative abundance (%) of taxonomic group with respect to total OTUs observed for community*.

b *Shannon diversity index*.

Non-metric multi-dimensional Scaling of a Bray-Curtis distance matrix demonstrated that some soil properties and/or spatial factors resulted in greater divergence within the Actinobacteria population (Figure 8). Axes 1 and 2 explained 71.8% of the Actinobacteria community variation. Concentrations of MP, MT, TN, AP, and ME were strongly associated with Axis 1 (loadings of −0.66, −0.63, 0.63, 0.58, and 0.56, respectively). MT, MP, ME, and TP were also strongly associated with Axis 2 (−0.59, −0.53, 0.47, and −0.40 respectively), and the pH (0.29, −0.15), OM (0.25, 0.25), and AK (0.26, −0.22) content had lower loadings than the other factors on both axes. AAC, MP, MT, and TP were correlated with YS1, YS3, YS4, and YS5 samples. OM, AP, TN, SD, and ME were correlated with YS2, YS7, YS8, YS9, and YS10 samples. An RDA analysis was employed to determine the influence of environmental factors on the Actinobacteria community (Figure 9). The first and second dimensions explained 42.2% of the total variance. The RDA analysis revealed that the Actinobacteria community compositions were related to multiple environmental factors, and other factors that were not studied in this paper.

## Discussion

Actinobacteria is one of the major phyla within the domain Bacteria. Because of the high diversity of members in this phylum, it is very difficult to develop a primer system that amplifies full-length, 16S rRNA gene sequences from all Actinobacteria. In spite of this, in the present study, it was possible to adopt indirect methods so that a larger number of full sequences could be screened from the bacterial 16S rDNA clone libraries. To simplify the screening process, we used 4 primers at the same time, and selected clones showed amplification bands (~270 and/or ~640 bp) for sequencing. Sequencing of the clone libraries clearly indicated that Actinobacteria DNA was primarily detected, with a false positive rate of 2.5%. The primer systems, Com2xf/Ac1186r/SC-Act235-aS-20/SC-Act878-aA-19, were suitable to screen for Actinobacteria in the 16S rDNA clone libraries.

**Figure 6:**
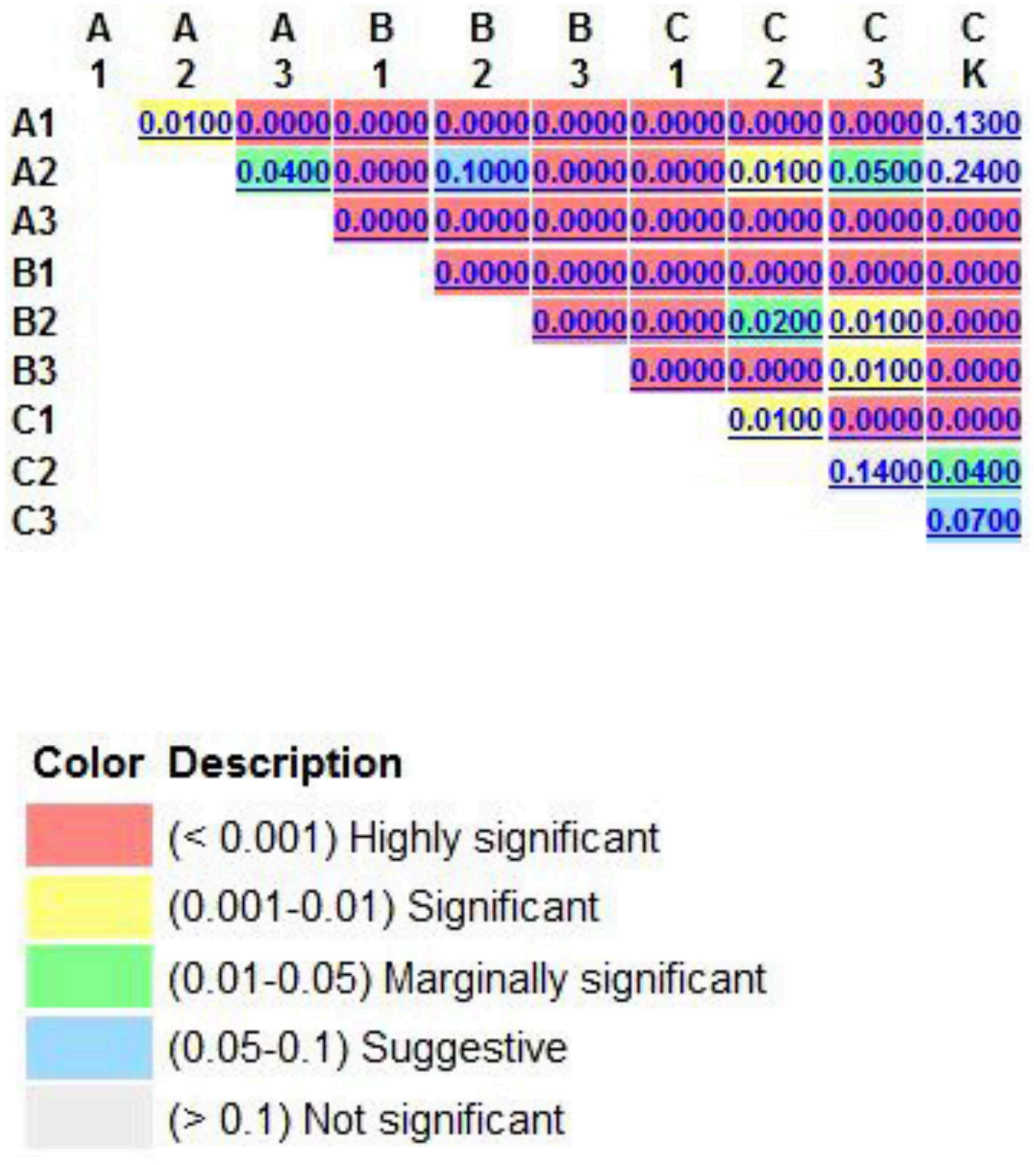
**The UniFrac significance were calculated by way of each pair of pretreatmental procotol, which tests whether each pair of environments differs from one another**. Air dried soil sample were treated by 120°C 1 h (A1); 2 h (A2); 3 h (A3); soil sample were treated by air drying processing 15 days (B1); 30 days (B2); 45 days (B3), soil sample were treated by 0.1% Polymyxin B Sulfate immersion 1 h (C1); 2 h (C2); 3 h (C3).

One of the aims of this study was to improve methods for detection and identification of Actinobacteria represented in 16S rDNA clone libraries derived from environmental samples. In the soil, the majority of bacterial 16S rDNA products were from non-Actinobacterial strains; Actinobacteria from the 16S rDNA clone library were relatively rare. In this study, we studied the effect of air-drying, heating, or 0.1% Polymyxin B Sulfate on analysis of Actinobacteria diversity using culture-independent methods. These pre-treatment methods for the culture and isolation of Actinobacteria have been suggested by several researchers (Demain and Davies, 1999; Seong et al., 2001; Jiang et al., 2010; Jensen et al., 2015; Sun et al., 2015). Employing pretreatments of soil by drying and heating has been shown to increase the number of actinomycetes that were isolated. In this study, when the total DNA of untreated soils was extracted, the colonies recovered was mainly from other orders of bacteria (Table 1). However, no matter which pretreatment method was applied, pretreatment significantly increased the numbers of Actinobacterial colonies (*P* < 0.01), while drastically reducing the numbers of other bacterial colonies (*P* < 0.01). The rarefaction analysis of OTUs at the 97% level suggested that the number of clones screened (500) was insufficient to cover the diversity of Actinobacteria and the data were rarefied (Figure 2). Therefore, in our analysis of Actinobacteria diversity in the Yanshan Mountains, we increased the number of clones screened to 1000. Data confirmed that the pretreatment of soil led to an increase in the detection of Actinobacteria taxa and access to a more genetically diverse community of Actinobacteria. At the same time, we found that each of the soil pretreatments could not only increase the detection rate of Actinobacteria, but showed a bias toward the detection of some groups of Actinobacteria. It provides a reference for the separation of the corresponding groups of Actinobacteria. Each of these treatment methods has both positive and negative aspects, in terms of their efficiency and ability to yield DNA extracts that truly represent the natural microbial community. Altogether, our results indicate that the Actinobacterial abundance and diversity that was detected might be affected by pretreatment procedures used to recover soil metagenomic DNA. Understanding these biases has become critical with the expansion of 16S rDNA technologies, which allow a more comprehensive investigation of specific microbial diversity. Our study confirms the pivotal importance of soil sample pretreatment in the DNA extraction procedure. It also emphasizes the need for thorough technical surveys to increase species richness per sequencing effort to be useful in microbial diversity studies. Consequently, we need to revisit our choice of pretreatment protocols to ensure that the DNA recovered from soil is not only of good quality, but also sufficiently representative in terms of richness and evenness of the Actinobacterial populations. In contrast to untreated soils, where Actinobacteria are believed to represent only about 3.6% of the total bacterial community, investigations of pretreated soils revealed that Actinobacterial 16S rRNA genes accounted for between 4.8 and 20.4% of the total community. The detected Actinobacteria were highly diverse (A1). Compared with other pretreatment methods, Actinobacteria diversity from methods A1 and A2 were not different with CK, as determined by the UniFrac significance test (0.13 and 0.24, respectively; Figure 6). Moreover, A1 yielded 102 clones with a detection rate of 20.4%, which was much higher than those found with the other pretreatment processes. Therefore, in order to gain accurate and representative phylogenetic information on Actinobacteria in the Yanshan Mountains, we chose the A1 soil pretreatment method: air drying of the soil sample, followed by exposure to 120°C for 1 h. With this method, we observed high Actinobacterial diversity in a relatively small number of clone libraries.

**Figure 7:**
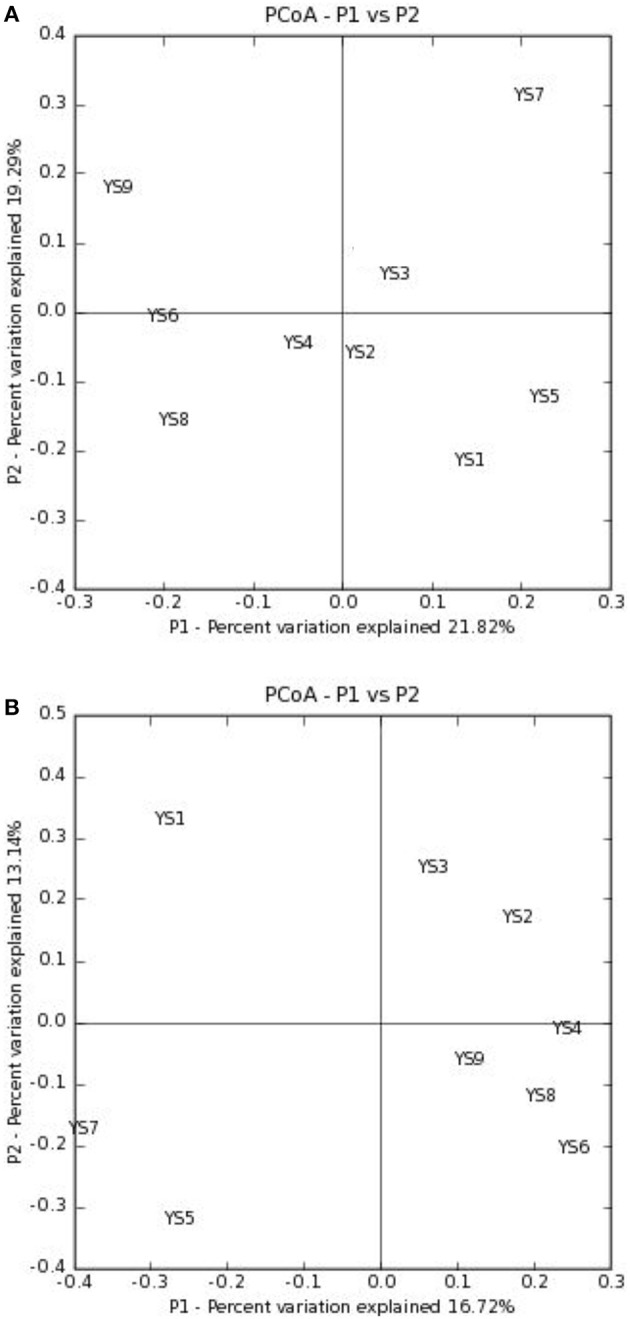
**PCoA plots are presented of the first two axes based on (A) weighted and (B) unweighted Unifrac distance matrices showing the quantitative and qualitative clustering of samples**.

**Figure 8:**
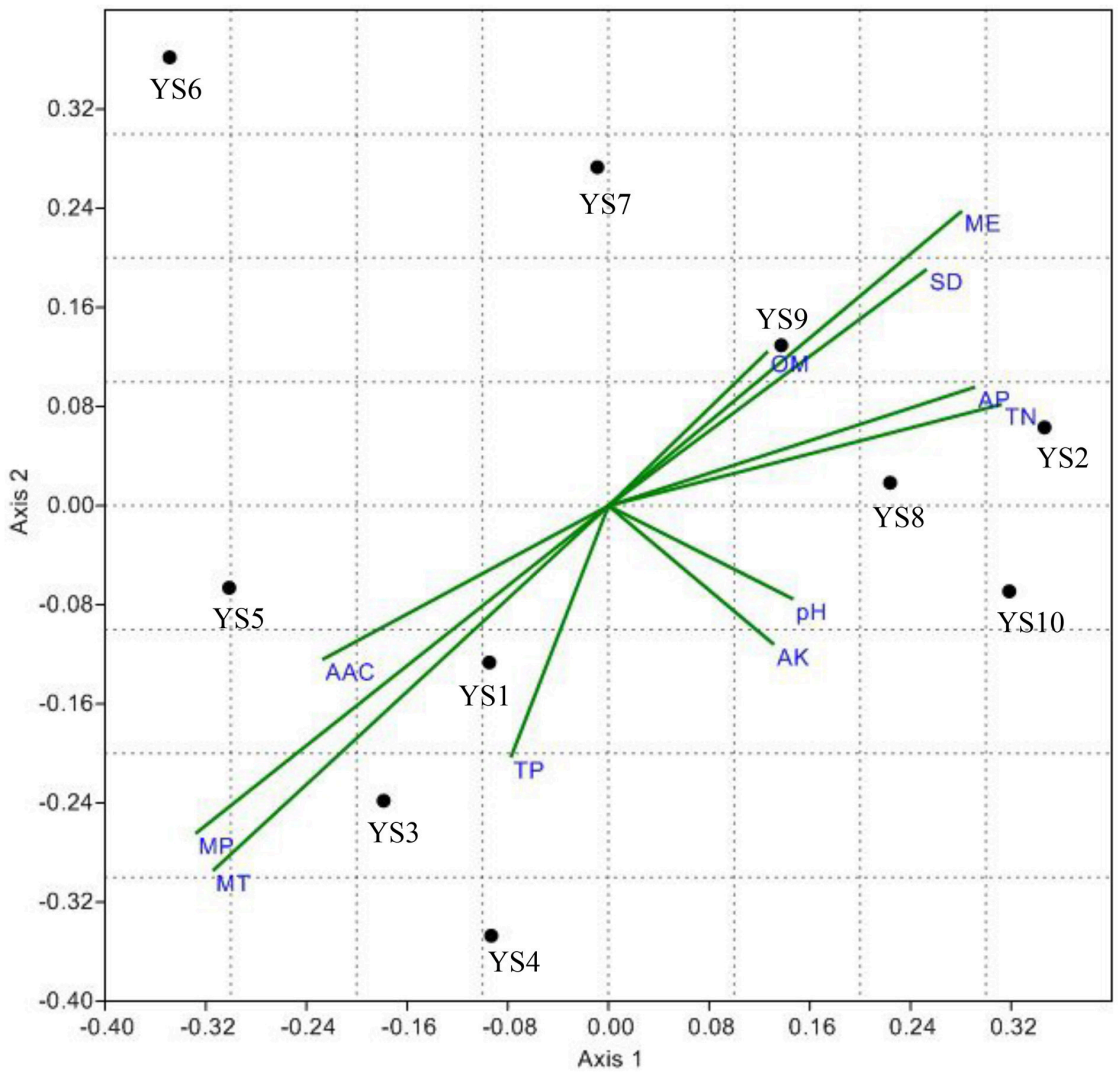
**Non-metric Multidimensional Scaling (NMDS) projection of a Bray–Curtis distance matrix showing the response of ***Actinobacteria*** communities to environmental vector**. Axis 1 explains 45.6% of variance, while Axis 2 describes an additional 26.2% of variance among samples. Environmental variables abbreviations are TN, total nitrogen; TP, total phosphorus; AP, available phosphorus; AK, available potassium; OM, organic matters; MT, mean temperature; MP, mean precipitation; ME, mean sea level elevation; SD, mean sunshine duration; AAC, mean active accumulated temperature.

**Figure 9:**
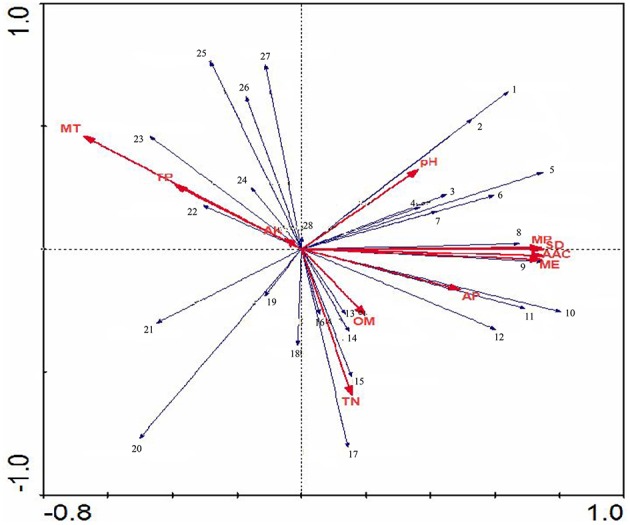
**The RDA ordination plot for the relationship between the family patterns of ***Actinobacteria*** community Clusters and environmental factors in the Yanshan mountains zone**. Correlations between pattern or environmentasl factors and RDA axes are represented by the length and angle of arrows.

Yanshan Mountains is a famous mountain range in north China, located at N 39°40′ ~ 42°10′, E 115°45′ ~ 119°50′ in the Inner Mongolia platform anteclise and subsidence zone. The eastern slope of the mountains has low mountains and hills, and lush vegetation, including shrubs, weeds, and a vast forest area. The western slope has low and medium mountains and sparse vegetation, including shrubs and grass. The Yanshan Mountains lie in an ecologically sensitive zone of north China near the Hu Huanyong Line (Hu, 1985). It is an ecosystem that has been adversely affected by forces of nature resulting in the destabilization of the balance of the living and non-living organisms in it and making it vulnerable to destruction. The ecosystem is facing changes due to climate change and destructive human activity, such as the mass cutting of trees. Living organisms interact with one another in an ecosystem in a cyclic manner; therefore, when one organism is destroyed, it affects the remaining organisms (Montoya et al., 2006).

Using a 16S rRNA gene clone library as a culture-independent method to survey the Actinobacterial community of Yanshan Mountains, we found that the overall diversity observed at the different stations was very high. The high number of novel Actinobacteria detected in the environmental samples is also significant. The Antibiotic Literature Database indicates that 57.8% of the known bioactive microbial products are produced by members of the class Actinobacteria. In this study, based on a comparison of signature nucleotides with higher taxa described by Zhi et al. (2009). we identified a total of 23 unclassified Actinobacteria, representing 2 novel orders, 10 novel suborders, and 39 novel families from Yanshan Mountains. It is reasonable to conclude that these new lineages may produce novel bioactive compounds, similar to other Actinobacteria. Clearly, the diversity of Actinobacteria greatly exceeds that predicted based on culture-based estimates, and this highlights the great biotechnological value in continuing efforts to isolate novel Actinobacteria genera. The genera *Conexibacter, Solirubrobacter, Microlunatus, Blastococcus*, and *Streptomyces* were common to all stations surveyed in this study. These groups are conserved in the Yanshan Mountains. Despite changing ecologies in the different stations, they were always present. Although members of the order Solirubrobacterales have not been extensively studied, recent studies have shown their ability to adapt and colonize different ecosystems, including fungal growing ant colonies (Ishak et al., 2011), spinach phyllosphere (Lopez-Velasco et al., 2011), desert, and Antarctic soil (Chong et al., 2012). Members of the genus *Blastococcus* were recovered from different latitudes and climates in dry and/or extreme conditions (Salazar et al., 2006), these microorganisms have the potential to colonize and alter stone and monument surfaces. *Microlunatus* spp. have been isolated from marine sediments (Yuan et al., 2014), a soil sample collected from Alu, an ancient cave (Cheng et al., 2013), and from conventional farming (Li et al., 2012). Some *Microlunatus* spp. have phosphorus-accumulating functions and phosphate uptake/release activities (Akar et al., 2005) in the enhanced biological phosphate removal (EBPR) process, and they are believed to play a pivotal role in phosphorus removal. The EBPR process is attracting interest for its potential use in phosphorus recycling (Hirota et al., 2010). In this study, some groups seemed to be more adaptive, based on their ability to survive in various environments. In contrast, there were unique genera identified in specific site: YS1, *Longispora, Propionibacterium*, and *Xylanimonas*; YS2, *Nocardia*; YS3, *Actinaurispora, Actinomadura, Actinomycetospora, Cryptosporangium, Humicoccus*, and *Phytohabitans*; YS4, *Actinocorallia, Actinospica, Hamadaea, Millisia*, and *Phycicoccus*; YS5, *Actinokineospora*; YS6, *Jiangella*; YS7, *Cellulomonas* and *Okibacterium*; YS8, *Rothia, Saccharothrix*, and *Terrabacter*; YS9, *Amycolatopsis, Cryobacterium, Knoellia, Nakamurella*, and *Rhodococcus*. Endemic taxa of these different stations reflect the Actinobacterial response to different environments.

The UniFrac analysis of the stations showed that the Actinobacterial compositions of YS2, YS3, YS4, YS6, YS8, YS9, and YS10 did not differ (*P* > 0.1). It is indeed “everything is everywhere, but the environment selects,” with no evident dispersal limitations on Actinobacteria, This theory suggests that each ecologically equivalent study site will have similar Actinobacterial communities due to near identical environmental variables, which eliminate environmental filtering as well as constant additions by the regional species pool. Conversely, Bissett et al. (2010) described a hypothesis of “wherever you go, that's where you are” implying that beyond strong environmental selection, other factors (i.e., dispersal or colonization limitations and evolutionary events) play a significant role in shaping microbial communities. Between YS7 and most other sites, there were significant different in Actinobacterial community composition, with YS4 and YS8 showing highly significant differences (*P* < 0.001). It has been suggested that microbial biogeographical patterns are shaped by environmental factors (Fierer, 2008). For instance, pH (Fierer and Jackson, 2006), temperature (Yergeau et al., 2007), and precipitation (Clark et al., 2009) have been found to be the best predictors of continent-scale patterns. It is also believed to be globally distributed by prevailing winds and community patterns are thought to result from barriers to dispersal, physiological requirements, resource availability, competition, or some combination thereof. However, Actinobacteria do not display a cosmopolitan distribution: their communities remain distinct not only over large geographical distances (Wawrik et al., 2007; Eisenlord et al., 2012) and seasonal differences (Cho et al., 2008), but also vary with local environmental factors^54^ and within a single sampling location (Abdulla and El-Shatoury, 2007; Van der Gucht et al., 2007). This work provides evidence that soil Actinobacterial communities exhibit regional biogeographic patterns, wherein community membership changes across the north-south distribution of Hu Huanyong Line. Stations YS1, YS3, YS4, and YS5 are located at the edge of the ecologically sensitive zone, the southern Yanshan Mountains, in the rain belt, and these sites are affected by the continental climate significantly. The climatic characteristics of AAC, MP, and MT and biogeochemical data of TP likely contributed to Actinobacterial communities at these stations. The ecological environments of other stations were not stable and fragile. It was clear that biogeochemical factors contributed more to Actinobacterial community structure than chemical factors. The stability of Actinobacterial communities in different ecological environments was largely correlated with biogeochemical factors and less with climate factors, such as *Streptomycelaceae* and pH, *Solirubrobacteraceae* and AP, *Propionibacteriaceae* and OM, *Geodermatophilaceae* and TN.

## Author contributions

HT, implementation of the experiment. XS, English check. XW, English check. HH, English check. XZ, sampling. LZ, designing experimental program.

### Conflict of interest statement

The authors declare that the research was conducted in the absence of any commercial or financial relationships that could be construed as a potential conflict of interest.
