# Supplementary material for: Environmental Controls Over Actinobacteria Communities in Ecological Sensitive Yanshan Mountains Zone
Source: Front Microbiol. 2016 Mar 22;7:343. doi: 10.3389/fmicb.2016.00343 (PMC4801888; doi:10.3389/fmicb.2016.00343)
Supplement: Supplementary file 1 [file Table1.DOC]

Legend:

Supplementary Figure 1 Analysis of the ‘Large Enough’ calculator to determine sample sufficient at the 97% similarity lever.

Supplementary Figure 2 The UniFrac significance were calculated by way of each pair of environments, which tests whether each pair of environments differs from one another. The calculated P-values are output in two separate data tables. In the first table, the values have been corrected for multiple comparisons by multiplying them by the number of comparisons that were made (the Bonferroni correction).

Supplementary Table 1. Basic properties of soil samples from Yanshan mountains zone. TN: total nitrogen; TP: total phosphorus; AP: available phosphorus; AK: available potassium; OM: organic matters

Supplementary Table 2. Basic spatial factor of sampling place from Yanshan mountains. MT: annual mean temperature; MP: annual mean precipitation; ME: mean sea level elevation; SD: annual mean sunshine duration; AAC: mean active accumulated temperature (>10℃)

Supplementary Figure 1


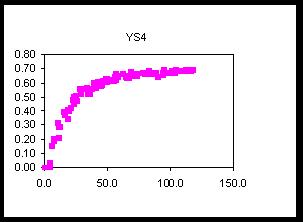


YS2


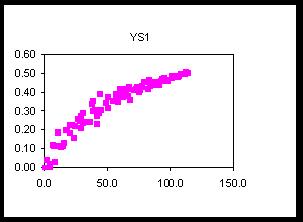


YS1


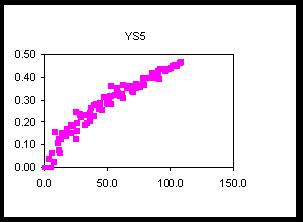


YS3


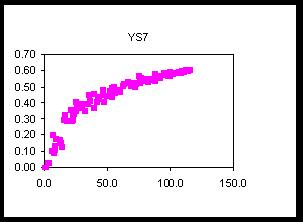


YS4


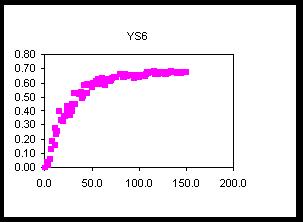

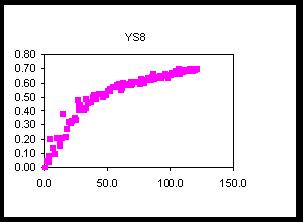


YS5


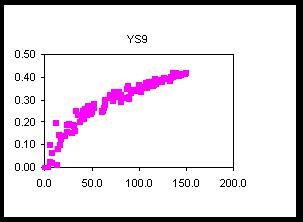


YS7


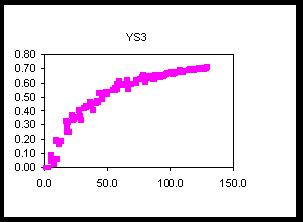


YS8


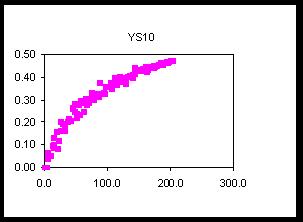


YS9


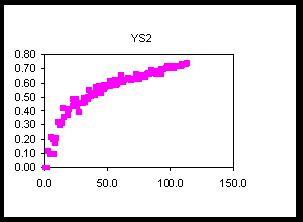


YS10

YS6


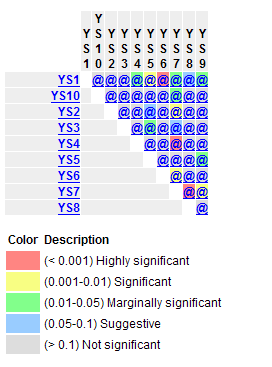

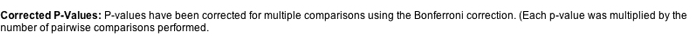


Supplementary Figure 2

Supplementary Table 1

| Sample | Soil texture | pH | OM | TN | TP | AP | AK |
| --- | --- | --- | --- | --- | --- | --- | --- |
|  |  |  | g/kg | g/kg | g/kg | mg/kg | mg/kg |
| YS1 | brown loam | 7.28 | 9.64 | 0.62 | 0.66 | 26.83 | 72.04 |
| YS2 | brown loam | 6.52 | 15.72 | 4.27 | 0.53 | 112.35 | 183.45 |
| YS3 | brown loam | 6.83 | 15.36 | 1.01 | 0.81 | 23.08 | 96.62 |
| YS4 | brown loam | 7.97 | 10.63 | 0.63 | 0.63 | 26.56 | 70.62 |
| YS5 | brown loam | 5.85 | 10.66 | 0.66 | 0.64 | 8.53 | 64.73 |
| YS6 | brown loam | 6.83 | 10.93 | 1.14 | 0.75 | 24.19 | 151.37 |
| YS7 | brown loam | 7.25 | 19.74 | 0.81 | 0.48 | 19.14 | 5.93 |
| YS8 | brown loam | 8.15 | 5.75 | 0.74 | 0.74 | 60.27 | 160.54 |
| YS9 | chestnut soil | 6.74 | 19.79 | 1.48 | 0.52 | 70.78 | 100.85 |
| YS10 | brown loam | 7.57 | 14.38 | 1.83 | 0.78 | 6.57 | 169.42 |

Supplementary Table 2

| Sample | Location(N;E) | MT (℃) | MP (mm) | SD (h) | ME (m) | AAC |
| --- | --- | --- | --- | --- | --- | --- |
| YS1 | 40.45^o^;116.71^o^ | 12.1±0.67 | 525.0±118 | 2778.9±122 | 351±5 | 25003±1762 |
| YS2 | 40.89^o^;117^o^ | 7.8±0.48 | 533.6±113 | 2694.1±139 | 455±3 | 32459±1812 |
| YS3 | 40.38^o^;117.17^o^ | 10.8±0.52 | 661.3±103 | 2667.2±114 | 387±7 | 36005±1324 |
| YS4 | 40.34^o^;118.09^o^ | 11.4±0.74 | 656.1±181 | 2482.6±204 | 70±6 | 41557±1693 |
| YS5 | 40.10^o^;118.40^o^ | 11.3±0.57 | 678.9±194 | 2443.6±197 | 149±5 | 41228±1515 |
| YS6 | 40.91^o^;118.77^o^ | 7.7±0.53 | 523.8±118 | 2728.3±132 | 631±3 | 32009±1957 |
| YS7 | 41.36^o^;119.59^o^ | 8.3±0.49 | 473.8±80 | 2843.3±118 | 468±8 | 34268±1425 |
| YS8 | 42.06^o^;118.29^o^ | 6.4±0.42 | 440.1±73 | 3025.7±125 | 686±4 | 31752±1482 |
| YS9 | 42.04^o^;117.83^o^ | 5.6±0.63 | 435.3±89 | 2711.1±126 | 938±5 | 26875±1759 |
| YS10 | 41.08^o^;116.09^o^ | 6.5±0.66 | 417.8±80 | 2737.9±192 | 1021±8 | 28196±1840 |
